# Ecofeminist heterotopias: gendered ecologies, memory, and ecological displacement in Elif Shafak’s *There Are Rivers in the Sky*

**DOI:** 10.3389/fsoc.2026.1915021

**Published:** 2026-09-17

**Authors:** Harini S, Patchainayagi S

**Affiliations:** School of Social Sciences and Languages, Vellore Institute of Technology, Chennai, Tamil Nadu, India

**Keywords:** ecofeminism, ecofeminist heterotopias, ecological displacement, Elif Shafak, heterotopias, hydrofeminism, social inequality

## Abstract

Ecofeminism reveals how ecological degradation and water insecurity have a profound impact on women and marginalized communities, emphasizing that ecology, power, displacement, and social inequality are deeply interconnected. The article proposes “ecofeminist heterotopias” as an analytical framework combining ecofeminism, Astrida Neimanis’ Hydrofeminism, and Michel Foucault’s concept of heterotopia to study how rivers function as gendered counter-spaces in the novel *There Are Rivers in the Sky* (2024). The research emphasizes that rivers in the novel do not simply function as ecological elements but are material, spatial, and relational sites where gendered violence, ecological precarity, displacement, and memory converge. Through a close analysis of the select text, the study examines the interconnected narratives of Arthur, Narin, and Zaleekhah traversing different ecofeminist heterotopias like the Thames, the Tigris, and hidden ‘ghost rivers’ across Victorian London, ancient Mesopotamia, contemporary Iraq and London. The rivers function simultaneously as archives of trauma, resistance, and cultural memory, illustrating the ways in which women and marginalized communities like the Yazidis, face the consequences of ecological destruction and forced displacement. By employing Astrida Neimanis’ Hydrofeminism and Foucault’s concept of heterotopia, the paper analyses how rivers as spaces that juxtapose incompatible temporalities and geographies material and spatial mediums while revealing the gendered facets of water crisis. This article posits that “ecofeminist heterotopias” provides a distinct analytical contribution to ecofeminist studies by depicting water not only as an ecological body but as an archive of feminist memory and a site of resistance against ecological and gendered violence.

## 1 Introduction

Ecofeminism is crucial to understanding how ecological degradation and unequal social relations intersect, especially in situations where access to and control over natural resources are structured by relations of gender, power, and marginalisation (Shiva, 1988; Plumwood, 1993; Gaard, 2001). Subsequent studies have further extended these concerns to address the issues of water scarcity, unequal distribution of environmental harm, displacement, and ecological justice (Bisht, 2009; Herrera Arango et al., 2022). In addition to these developments, an important area of scholarship, as seen in the works of Thompson (2016) and Acevedo-Guerrero et al. (2024), is the material and spatial aspects of ecological experience, with particular focus on the role of water in influencing embodied, historical and gendered connections. These perspectives are especially valuable and relevant to literary texts, where ecological sites function not only as symbols or settings, but as spaces where historical memories, social relations, and power dynamics are negotiated. The work *There Are Rivers in the Sky* (2024) by Elif Shafak is focused on the intersection of these themes. Spanning different timelines across ancient Mesopotamia, Victorian London, and contemporary Middle East and London, the text is centred around the interconnected narratives of Arthur, Narin, and Zaleekhah. It depicts how a recurring drop of water connects all three stories across different historical periods, making rains, rivers and other watery formations primary to the literary text’s narrative structure (Shafak, 2024). Water hence functions as more than a backdrop to human experience, connecting bodies, histories, memories, and different geographies across time. In the novel, the rivers portrayed, i.e., the Tigris, the Thames, and the “ghost” or buried rivers, although geographically distinct, are united through the cyclical journey of water, carrying histories of civilisations, personal narratives, collective memories, displacement, loss, and survival.

The aquatic spaces in the text are situated in sites that are marked by violence and ecological transformation. Narin’s narrative is grounded in the historical displacement and violence faced by the Yazidi community, while Arthur and Zaleekhah’s stories are also marked by personal loss, displacement, altered environmental spaces, and the consequences of human intervention in ecological spaces. These narratives are not disparate experiences but connect ecological degradation along with the traumatic histories of marginalized communities. The literary text constantly represents human suffering and ecological degradation in relation to one another, bringing bodies, water, history, and place into relation. These representations make the text particularly relevant in analysing how ecological spaces simultaneously sustain life, preserve memory, function as sites of violence, and reflect unequal relations of power. As a relatively recent text, scholarly reception surrounding the novel is still emerging, examining several narrative aspects of the work. Scholarship surrounding the text focuses on issues of identity, cultural belonging, and cultural hybridity (Muzaffar, 2025; Kanwal et al., 2025; Rehman et al., 2025). These studies address the literary work’s interaction with fluid identities, cultural difference, and the relationship between identity, place, and history. However, the relationship between watery materiality, contested spaces, and gendered ecological connections remains insufficiently explored.

This study addresses this analytical space through the concept of ecofeminist heterotopias. The concept brings ecofeminism into dialogue with Astrida Neimanis’ hydrofeminist understanding of the material relation between bodies and water and Foucault’s concept of heterotopia. This paper therefore studies how *There Are Rivers in The Sky* constructs rivers and waterways as material, relational, and spatial formations through which gender, memory, displacement, and power intersect. Through close textual analysis of the interconnected narratives of Arthur, Narin, and Zaleekhah, the paper investigates how water connects bodies and histories through different temporal and geographical contexts with certain ecological sites, especially river and waterways, accumulate memories of transformation, loss, and survival. Through the concept of ecofeminist heterotopias, the article contributes to sociological discussions on environmental, gendered, and hierarchical inequality by demonstrating how literary representations of ecological space can illustrate the spatial and embodied structures of power, collective memory, displacement, and marginalization. As a result, the study brings literary perspectives into conversation with sociological debates on environmental inequality and the unjust social consequences of ecological transformation.

## 2 Literature review

Ecofeminist scholarship consistently studies the relationship between environmental exploitation and unequal structures of gender and power. Foundational texts on ecofeminism have established that the domination of nature and marginalisation of women and minorities stem from the same root cause, i.e., hierarchical power structures (Shiva, 1988; Plumwood, 1993). Shiva’s (1988) criticism on development, scientific reductionism, and resource extraction foregrounds the unequal effects of environmental transformation, particularly on communities whose everyday life depends directly on land and water. At the same time, Plumwood (1993), identifies and critiques the dualistic structures through which Western systems of thought separate and categorise culture/nature, reason/emotion/ masculine/feminine into hierarchies. These approaches remain central to the current study as they offer a basis for examining ecological spaces as sites structured by historically unequal power dynamics.

Succeeding ecofeminist scholarship has further explored the relation between marginalized sections and nature by focusing on specific political, material, and social conditions through which environmental inequality is experienced. Gaard (2001), for instance, demonstrates the relevance of ecofeminist analysis regarding environmental politics and resource use. Further studies depict the impact of water insecurity on women and marginalized communities, highlighting that “water-related issues are not gender-neutral” (Oluwasanya et al., 2025; Bisht, 2009). Similarly, Herrera Arango et al. (2022) posits how acute water scarcity and environmental degradation are not isolated events but must account for the unequal access to land, resources, and rights throughout history. The article observes the impact of water privatization in affecting both the environment and marginalized communities, noting how systemic exclusion of these communities from accessing water bodies makes it difficult for them to perform day to day activities. On the other hand, Ibarra (2025) also studies privatization of water bodies in Chile shifting attention from water as a passive resource towards the cultural, social, and emotional relationships through which communities understand and defend rivers. The article depicts the river not merely as a resource but as a living being to which communities, especially women, have a deep spiritual, cultural, and emotional connection.

Collectively, existing scholarship places emphasis on how environmental harm is not experienced uniformly. These studies focus on environmental conflicts, developmental projects, water governance, and social movements, foregrounding the complex physical as well as relational connection between environmental spaces, especially water bodies, and human beings. Literary texts engage with these issues differently, representing and restructuring relationships between water, bodies, memory, and place through narrative form, character, and spatial imagination. In *There Are Rivers in The Sky*, issues of displacement, cultural loss, vulnerability and ecological transformation are connected through rivers and waterways that connects different bodies, historical periods, and locations. A literary analysis therefore examines how the literary text illustrates environmental spaces as sites where physical spaces, social hierarchies, and collective memories converge. This adds to existing research by focusing on the cultural and narrative forms through which its interconnected social meanings are imagined and articulated.

The importance of water within ecofeminist studies has resulted in the conception of a connected body of work that focuses on water as not merely a resource but as an embodied, material presence that flows through, connects, and sustains all physical bodies and environments. This study perceives the river through the lens of Hydrofeminism (2012), a concept introduced by Neimanis, who posits that water is not just a resource for human use but is deeply interconnected with humans, connecting humans to other lifeforms “For us humans, the flow and flush of waters sustain our own bodies, but also connect them to other bodies, to other worlds beyond our human selves.” (Neimanis, 2017) The concept of Hydrofeminism lies at the intersection of ecofeminism and blue humanities, focusing on water as an agentic being influencing human lives. As a result, water and its associated bodies are not isolated structures, but form an interconnected relationship with humans, making humans equally accountable and responsible towards the environment. Hydrofeminism views water as both a physical substance and also a way of understanding the world. Recent scholarship exhibits how hydrofeminist thought has evolved across different fields. In literary studies, a hydrofeminist study of Hall’s (2021) *Burntcoat* depicts how watery imagery disrupts and destabilises the fixed distinctions between the body and the environment (Chmielewska, 2024). Further scholarship reveals how application of hydrofeminist perspectives in illustrating gender, social exclusion, and water in cultural texts, illustrates the role of aquatic spaces in simultaneously sustaining, regulating, and constraining marginalised subjects. These studies establish hydrofeminism as a means of studying the material, embodied, and relational dimensions of water and waterways.

Hydrofeminism does not, however, does not sufficiently acknowledge how or why certain ecological spaces become sites of exclusion, displacement, control, memory, or social conflict. This is especially relevant in the analysis of *There Are Rivers in The Sky*, where rivers are not merely materially connected to bodies and histories but are also spaces whose meanings and existence are transformed and defined through urbanisation, displacement, cultural memory, and social conflicts. In order to study the spaces where the relationship between water and humans are formed, the concept of heterotopia by Michel Foucault becomes important here. Foucault first introduces the concept of heterotopia in contrast to utopia. While utopia refers to unreal, idealized spaces that reflect present society in a perfected form, Foucault describes heterotopias as real places that act as counter-sites, where these real sites are contested, represented, and inverted (Foucault and Miskowiec, 1986). Foucault’s framework of Heterotopia is grounded in the concept of “other spaces” that exist and function outside of real space where hegemonic norms exist (Foucault and Miskowiec, 1986). These spaces are “inverted” forms of real, physical spaces and constantly challenge the notion of human autonomy and self-sufficiency from other spaces and objects. Foucault’s concept is useful in literary and cultural analysis, as it enables an understanding of how certain spaces are not passive, geographical locations, but are transformed by power, access, memory, and social relations. In the context of hydrofeminism, not all waterways become a heterotopic space. Such an analysis requires attention to specific ways in which a text constructs a space, or a riverine space in this context, as a heterotopic space.

Scholarly engagement with *There Are Rivers in The Sky* is still emerging and has approached the text from several distinct perspectives. Bădulescu (2024) examines the text’s transhistorical narrative structure, historical and cultural imagery, as well as the interconnected significance of the Thames and the Tigris. Other studies focus primarily on the application of Third Space by Homi K. Bhabha to analyse transnational cultural negotiation and identity formation in the text (Kanwal et al., 2025; Rehman et al., 2025), while Muzaffar (2025) studies water as a non-human force with agency through the lens of posthumanism. However, the intersection of water’s material interconnectedness with the spatial structure of ecological and social inequality is still not thoroughly understood. Specifically, there is a need to study how riverine sites, i.e., rivers along with its associated ecosystem and landscapes, function as hydrofeminist archives of ecological memory combined with heterotopic spaces that reflect narratives of resistance, displacement, socio-political disputes and ecological risk. The paper contributes to the field by integrating both frameworks to propose the concept of ecofeminist heterotopias. This framework offers a nuanced analysis of how rivers, ecology and gender intersect in the novel *There Are Rivers in the Sky*, especially in relation to migration, displacement, and ecological precarity. The study also looks at the necessity of this framework especially in contemporary situations where climate change, water crisis, and ecological fragility highlight the importance of a framework that does not merely focus on human subjectivity but is rooted in mutual care and understanding.

## 3 Theoretical framework

The research builds upon Ecofeminism as the primary theoretical framework to study how environmental degradation and gendered inequality are connected in the text *There Are Rivers in the Sky* by Shafak (2024). Ecofeminism is crucial in understanding how ecological crises are rooted within the frameworks of patriarchy, capitalism, power, and inequality rather than existing as isolated environmental issues. These socio-political forces unevenly impact access to environmental resources like water, ecological degradation, and forced displacement among different social groups. An ecofeminist reading therefore enables this study to examine how ecological degradation becomes differentiated through gender, social hierarchy, and power.

From the broad framework of Ecofeminism, the research incorporates the concept of Hydrofeminism as formulated by Neimanis (2012, 2017). Neimanis’ work looks at water and water bodies from a material and relational standpoint, highlighting how bodies are constructed, shaped, and interconnected with the environment, particularly with water. The concept is influenced by Stacy Alaimo’s concept of Transcorporeality (Neimanis, 2017), as it reimagines bodies as fluid and relative, emphasizing on the deep, inseparable bond between human activities and the environment. Neimanis states that water should not be considered as a mere metaphor but a physical, material medium through which bodies, environments, and histories are shaped and connected.

We could also tell the story this way: water connects bodies across times and spaces, through various complex movements and cycles to other bodies and beings in diverse exchanges, gifts, thefts, and forsaking. We could think of this work of water as flow, or more specifically, as a logic of connection or communication (Neimanis, 2012).

By viewing rivers and water bodies as relational mediums that carry memory, vulnerability, and ecological histories, hydrofeminism challenges Western ideologies of anthropocentrism, discrete individualism, and phallagocentrism, which looks at humans as individually separate, superior, individuals and natural resources like water are merely static mediums to fulfil human needs (Bezan and Neimanis, 2022). In her concept, Neimanis further discusses the term “hydrocommons” as a central component of the concept hydrofeminism. Hydrocommons defines water as a planetary relation connecting human and non-human life together. She proposes “hydrocommons” as a framework for understanding the shared, embodied, and more-than-human interconnection of bodies of water (Neimanis, 2009). In order to understand how such interconnections are or organised within certain spaces or ecological sites, thereby embodying exclusion, access, displacement, and the collection of histories, Foucault’s concept of Heterotopia is employed.

Foucault and Miskowiec’s (1986) concept of Heterotopia is utilised to understand how spatial dimensions’ influence and shape the intersection of ecological and relational structures. Foucault’s heterotopia helps understand how environmental and social spaces evolve through constant interaction with each other. While Neimanis’ concept emphasizes the fluidity of bodies and their relations to water, Foucault’s heterotopia emphasizes on the continuum of space. Rather than represent a physical space as a static, singular entity, heterotopia observes how the physical space continues to exist and expand beyond its literal meaning, including different temporalities, meanings, rituals etc., depicting how spaces function as sites of simultaneity. In the context of this study, rivers and other related environments function as heterotopic spaces as they link different places and multiple temporalities reflecting structures of exclusion, mobility, and resistance. The concept provides a spatial lens of ecological sites, highlighting how ecological occurrences are not only material and relational but also spatially constructed.

The concept of heterotopia and the ecofeminist theory of hydrofeminism are synthesized through Ecofeminist Heterotopias. The relevance of these concepts rests in facilitating an integrated reading of ecological spaces that cannot be understood exclusively as material environments, social spaces, or symbolic settings. This framework studies ecological spaces as material, relational, and spatially contested formations simultaneously. While hydrofeminism reinforces the relationship between water, human bodies, and ecological systems, heterotopia provides a spatial framework to comprehend how these ecological spaces simultaneously function as sites of crisis, exclusion, displacement, memory, and resistance. The framework of ecofeminist heterotopias allows these ecological spaces to be read as dynamic sites where environmental degradation, gendered inequality, socio-political hierarchies and memory intersect. As a result, ecofeminist heterotopias become sites where marginalization as well as ecological harm become visible through embodied and spatial experiences.

In the analysis of the literary text, this framework allows to understand how environmental spaces simultaneously sustains life and also reflect structures of power, displacement, and inequality. In the novel rivers, ghost streams or hidden rivers, and dams become heterotopic formations of the environment, preserving histories of violence, displacement, survival, and resistance across different timelines. This framework allows for an integrated reading of water as a material, a relational, and spatial formation resulting in a multidimensional approach to study how gender, environment, and displacement interact and intersect.

## 4 Materials and methods

The paper uses a qualitative, interpretative approach, utilizing a close textual reading as the primary method to analyse the text *There Are Rivers in the Sky* by Shafak (2024). The interdisciplinary framework that guides the analysis employs ecofeminism, which is extended through the concept of hydrofeminism, and examined spatially through heterotopia. Through this approach, the paper studies how ecological, gendered, and spatial dynamics are reflected and connected within the narrative. The study places the analysis of the text within broader socio-ecological contexts, initiating conversation between literary representation and contemporary issues such as climate change, water politics, forced displacement, and environmental justice.

## 5 Discussion

Water is the primary connecting structure in Elif Shafak’s *There Are Rivers in The Sky*. While the novel evokes water in multiple forms, the three rivers that the novel explicitly focuses on, i.e., the river Tigris, the river Thames, and the buried or “ghost” rivers referenced in the literary text. The latter is not one river, but a collection of various streams, rivers as well as other water bodies that were buried over time due to urbanization and the necessity for space. These water bodies are referred to as “ghost” streams or “ghost” rivers, emphasizing their paradoxical condition where these rivers have disappeared from public view and collective memory and yet continue to exist even today.

‘*The Bièvre – it’s buried under the French capital, and has been for a long time. It was an important waterway until the nineteenth century, when it became heavily polluted. They covered it over and basically forgot about it. The tourists who walk around Paris today admire the Seine, but they don’t realize there’s another river flowing beneath their feet*.’ (Shafak, 2024, p. 163)

The rivers chosen in the text are connected through various meanings and values. The Thames and Tigris are geographically unrelated and are shaped by different historical contexts. However, the novel connects through the cyclical movement of water. This interconnected structure is established from the beginning through the journey of a single drop of water, “As ripples of heat rise into the air, the raindrop will slowly evaporate. But it won’t disappear. Sooner or later, that tiny, translucent bead of water will ascend back to the blue skies. Once there, it will bide its time, waiting to return to this troubled earth again … and again” (Shafak, 2024, p. 18). Water consequently unsettles the boundaries between different places and historical time periods. Rivers are fluid bridges – channels of communication between separate worlds. They link one bank to the other, the past to the future, the spring to the delta … (Shafak, 2024, p. 432). This reinforces Neimanis’ statement, “river delta or backyard pond—all of these bodies are necessarily brought into being by another body of water that dissolves, partially or completely, to water the bodies that will follow” (2012, 87). The novel’s rivers are therefore not disparate bodies, but are set in a larger hydrological cycle where histories, bodies, and environments are brought into relation.

The narratives of Narin, Arthur, and Zaleekhah, the central characters in the novel are connected through these rivers. The narration itself is structured in the shape of a water molecule H_2_O, with every chapter containing Arthur’s narrative labelled O while Narin and Zaleekhah’s narratives are labelled H, symbolizing that their lives bend and travel across time to form a connection that culminates in the final chapter, labelled H_2_O. This deliberate structure reinforces the idea that the separate lives of the characters are connected through water. This relationship does not imply that the characters’ experience with ecological spaces are similar to one another. Their relationships with riverine spaces and other water bodies are differentiated by class, gender, historical violence, displacement, and social position. This combination of material interrelation and unequal social experience facilitates the analysis of the novel through ecofeminist heterotopias.

### 5.1 Hydrofeminism in *There Are Rivers in the Sky*

In the novel *There Are Rivers in the Sky*, rivers play a pivotal role in shaping characters’ experiences, identities, and lives. Hydrofeminism emphasizes how water is a conduit of memory, carrying histories and cultures. Rather than functioning merely as a metaphor for fluidity or memory, water in the novel materially shapes the conditions in which the characters live. This is particularly evident in Narin’s relationship with the Tigris. For her and her community, the Yazidis, the rivers is profoundly important in shaping their ancestry, cultural memory, and everyday life. However, the narrative also depicts how the vulnerability of a community and that of its ecological surrounding can become entangled during periods of violence and displacement.

Narin’s story is set against the historical persecution and displacement of the Yazidi community. In 2014, they were the targets of a genocide by the ISIS or ISIL, resulting in a severe drop in their population and widespread displacement. Reports by the European Union Agency for Asylum (EUAA) state that,

Thousands of Yazidis were killed or abducted by ISIL during its rule. Yazidi women and girls suffered rape, torture, and slavery under ISIL captivity and pregnant survivors of sexual violence were highly vulnerable to various forms of violence. Younger boys faced indoctrination, beatings and torture by ISIL and commonly became child soldiers. (EUAA, 2024)

Shafak incorporates this history into Narin’s narrative while also focusing on the destruction and transformation of ecological spaces, highlighting how this violence and oppression is not a singular event against humans but also has an adverse impact on the environment and surrounding space. In the novel, Narin’s monologue, “an enemy who poisons water and torches trees – what will they do to human beings?” (Shafak, 2024, p. 345) establishes that the environment, especially water, is not a neutral backdrop, but is inextricably linked with bodily existence, collective memory, and vulnerability of the community. Shafak’s positioning of water as a relational body that is deeply interconnected with the human body, reflects Neimanis’ idea of hydrocommons. Furthermore, the novel traces a pattern of violence beginning with the destruction of riverine bodies which gradually affects humans, especially women.

‘*Water …’ murmurs the captive. For a moment they think he is asking for a drink, but then they hear him say, ‘It is a gift from the gods; it gives us life, joy and riches aplenty. But you, my lord, turned it into a deadly weapon. No more fish left in the River Ulai: you choked it with so many corpses that it flows the colour of dyed-red wool. First the drought, then the famine. My king, your subjects are starving. The plains of Susa are strewn with the dead and the dying. Now, I hear, you will do the same in Castrum Kefa …*’ (Shafak, 2024, p. 15)

This reflects how water is transformed from a source of life into a tool of violence. Its material condition changes through human action, while this ecological transformation simultaneously impacts the bodies and communities dependent upon it. Through the lens of hydrofeminism, the passage challenges the distinction between social and environmental violence. The river is not merely a witness, but becomes implicated in the violence experienced by the victims.

Narin’s relationship with the Tigris also illuminates how water carries cultural and collective memory. Through her grandmother’s stories, her community’s religious traditions, and the landscape which is at the risk of being separated from her due to displacement, Narin’s connection with the river embodies a deep bond with nature. The construction of the Ilisu Dam intensifies this vulnerability by threatening to alter both the physical environment and the historical relationships embedded within it. When Narin’s grandmother delays leaving her home, her attachment is directed not simply towards property but towards a riverine landscape that contains cultural and ancestral significance:

*Grandma always says she will be one of the last to go. She cannot abandon her pistachio trees. But primarily it is the river she cannot part from. Every so often she walks to these shores, lamenting that the ground under her feet is about to be flooded, thousands of years of history gone, knowing that these are her last prayers to the Tigris*. (Shafak, 2024, p. 38)

Meanwhile, Zaleekhah’s narrative, set in contemporary London, reflects the ongoing climate crisis and the vulnerability of water bodies. The narrative focuses on particular instances of ancient rivers which have been permanently buried in the process of development and construction of cities. Termed as ‘ghost streams’ or ‘ghost rivers’, they refer to “…waterways that previously existed but, as urban areas built up, were either buried below the surface or filled in to support development.” (Napieralski et al., 2023; Napieralski, 2024). Shafak references to several ghost streams that still exist today, buried under roads, buildings and other urban structures throughout the world. “A legendary river, then an open sewer, then an aimless canal, then an open sewer again and, eventually, forgotten by almost everyone. Still alive, though. A watery spectre that refuses to die” (Shafak, 2024, pp. 194–195). Zaleekhah’s work as a hydrologist directly aligns with hydrofeminism, as her work is not only rooted in scientific theories but also encompasses the concept of aquatic memories and the discovery and reclamation of ghost rivers, aligning technical knowledge with hydrofeminist practices that foreground memory, care, and accountability. Her approach towards water conservation and protection is rooted in a hydrofeminist framework that is not limited to technical mastery but also considers water conservation as a deeply personal responsibility of every individual.

By presenting rivers as sites that simultaneously sustain life and face the repercussions of human exploitation and ecological violence, the text depicts that women and marginalized communities face disproportionate experiences of environmental insecurity and systemic exclusion. Through the portrayal of Narin, Zaleekhah and other characters, the text demonstrates how hydrofeminist relations are constructed by gendered ecological inequality and persecution of minorities.

### 5.2 Rivers as a heterotopic space

The novel’s structure reinforces rivers as heterotopic sites where different time periods, histories, and social orders merge and coexist. Foucault’s description of heterotopic sites as real spaces that invert dominant order is reflected through the rivers Thames and Tigris. Rather than treating the Tigris, the Thames, or ghost rivers as heterotopias simply because they are unusual or symbolically complex spaces, this reading focuses on the specific heterotopic qualities they acquire in the narrative. These rivers function as alternates spaces that exists beyond the boundaries of temporal and geographical distance, connecting ancient Mesopotamia, Victorian England, and the contemporary world. The non-linear structure of the narrative, which constantly shifts between time periods while being anchored by water, creates a layered space that allows different time periods and histories to co-exist within the same hydrological space.

The rivers portrayed in the novel could be read as heterotopic spaces that embody all six principles of heterotopia as outlined by Foucault. In the first principle, Foucault states that every culture consists of heterotopias but they exist in varied forms (Foucault and Miskowiec, 1986). The rivers become heterotopic sites for individuals in crisis or on the margins of society. Arthur is born into poverty at the banks of the dirty river Thames, earning him the name, “‘King Arthur of the Sewers and Slums!’ And that is how the boy born on a secluded stretch of the Chelsea waterfront, under the low-lying branches of a bankside oak tree, will someday come to be known to all. A child of the river he is, and so he will remain all his life” (Shafak, 2024, p. 29). Similarly, the river Tigris embodies a ‘crisis heterotopia’ for Narin and the Yazidi community, who are shunned and persecuted for their faith, while in Zaleekhah’s narrative, modern-day Thames becomes a heterotopia of deviation as she moves into a houseboat to escape her ruined marriage. The second principle states that as society evolves, the function of the heterotopia also changes. In the text river Thames is regarded as a “zombie” that was revived from the dead (Shafak, 2024, p. 114). Zaleekhah and Arthur’s narratives reveal how, during the Victorian era, Thames was a dumping ground for industrial effluents and an open sewer carrying the city’s waste. The river is later revived but still carries the city’s waste, demonstrating how the function of the riverine space constantly fluctuates as time and society evolves. In the past, the polluted river was the home slums and ‘toshers’ or scavengers (Shafak, 2024, p. 21). However, contemporary Thames has been completely transformed housing London’s affluent people, highlighting how the river as a heterotopic space has evolved according to the changing time periods and societal standards. She recalls reading …The river, once so polluted that it was called the ‘monster soup’, nowadays offers a coveted view for which plenty are happy to pay excessive premiums (Shafak, 2024, p.66).

Shafak’s depiction of the Thames, the Tigris, and numerous ghost streams which were once vibrant sites of culture and history but now buried and hidden due to civilization and progress depicts how the function of these heterotopic sites keep evolving constantly. The next principle of heterotopia claims that, “The heterotopia is capable of juxtaposing in a single real place several spaces, several sites that are in themselves incompatible” (Foucault and Miskowiec, 1986). In the text, both the rivers and the droplet of water that travels throughout the novel across timelines in its cyclical journey perfectly embody this principle. The novel reveals the rivers as a heterotopic space where both the sacred nature of the Tigris and profane act of poisoning the river, the past and present of the rise and fall of empires, the progress of science and technology through the construction of dams on the river and slow ecological violence, and the home, culture of the Yazidis and their exile, all juxtapose to reveal rivers as heterotopic spaces where incompatible worlds coexist. Shafak narrates how rivers and its surrounding landscapes are carriers of memory, culture, ancestry, and communal identity which resonates with Foucault’s concept of Heterotopia. Shafak uses a single drop of water, the rivers Tigris, Thames, and the ghost rivers to connect different time periods in history, i.e., Victorian London, ancient Mesopotamia, and contemporary Iraq, transforming these rivers into heterochronies, or spaces that break ordinary time and transform into temporal crossings connecting past and present. This forms the fourth principle of heterotopia which describes how heterotopias are connected to time, often accumulating or fragmenting it.

Foucault talks about access and entry to heterotopias in his fifth principle. He claims that “Heterotopias always presuppose a system of opening and closing that both isolates them and makes them penetrable. In general, the heterotopic site is not freely accessible like a public place” (Foucault and Miskowiec, 1986). In the text, Narin’s family and neighbours face the risk of displacement and erasure of their culture due to the construction of the Ilisu Dam, highlighting how access to riverine spaces is imbalanced, shaped by socio-political and economic structures. In the context of the text, riverine spaces exemplify the fifth principle by highlighting how marginalized communities, especially women experience limited access to water due to displacement and conflict. This is further observed in Narin’s narration, where the river is a site of spiritual purification and baptism using holy water. However, this ceremony is interrupted due to ongoing construction of the dam Ilisu and remains unfulfilled due to the conflict with the ISIS.

‘But what about my baptism? We were going to visit the Valley of Lalish next week. I prepared my dress.’… ‘I know, sweetheart, but … this place doesn’t feel safe.’ He clears his throat, realizing he should not alarm the child unnecessarily. ‘I do not expect these fanatics to do anything more extreme (Shafak, 2024, p. 346).

Finally, the sixth and last principle states that all heterotopic spaces are relational to all other spaces that remains. They either create spaces of illusion “that exposes every real space” (Foucault and Miskowiec, 1986) or create spaces that compensate or make up for the disorder of these real spaces. In the novel, the Thames of the present time is reclaimed from its polluted past and is branded as a clean river housing London’s wealthy communities. However, this is only a space of illusion created by the heterotopic river, as it hides the city’s continued pollution. “Now considered one of the most handsome and cleanest natural streams in the world, it nevertheless continues to ingest the waste of a city of millions” (Shafak, 2024, p. 114). The river Thames becomes a space of compensation for Arthur by allowing him to escape the rigid class system through intellectual discovery. The river Tigris also exist as spaces of compensation and gendered ecological connection to Narin, by providing an ancient, spiritual continuity that compensates for the violence of genocide by the ISIS.

In *There Are Rivers in the Sky*, the interactions between the characters and riverine bodies reveal its heterotopic quality. The river becomes at once both familiar and distant, a site of belonging and displacement, continuity and rupture. The novel frames rivers and by extension ecological sites as a heterotopic space, emphasizing how the environment and people are together structured by overlapping timelines and hierarchies. These heterotopic spaces disrupt the illusion of normalcy and order in real spaces by constantly evolving over time based on socio-political conditions of a place. The six principles of heterotopia reveal how rivers are not passive entities, but active, alive spaces that are pivotal, politically and affectively charged counter-narratives of memory, resistance, and precarity. By combining hydrofeminism with heterotopia, rivers become embodied, porous, gendered and relational sites, revealing that the vulnerability of rivers parallels the fragility of women and marginalized communities and closely interlinked to one another.

### 5.3 Ecofeminist heterotopias and the intersections of gender, ecology, and displacement

The concept of ecofeminist heterotopias emerges from the intersection of these material and spatial concerns. It does not simply combine hydrofeminism and heterotopia under a new label. Rather, the concept identifies ecological sites in which three dimensions operate simultaneously, i.e., the material interconnectedness of bodies and environments; the spatial convergence of different histories and social meanings; and the unequal structures of gender, class, displacement, and power through which ecological vulnerability is experienced. Ecofeminism is essential to this formulation because it directs attention to the fact that environmental harm is neither socially neutral nor experienced uniformly (Plumwood, 1993; Gaard, 2001). Hydrofeminism foregrounds material and relational continuity, while heterotopia explains spatial contradiction and simultaneity. Ecofeminist heterotopias therefore provides a framework for examining how ecological risk, gendered violence, and social inequality are jointly experienced.

The issues discussed in *There Are Rivers in the Sky* include water shortage, pollution, negative effects of dam building, displacement, histories of ghost rivers and artificial poisoning of rivers, which reflect how they unfairly affect women, marginalized communities, and displaced people, highlighting how ecological insecurity is always socially uneven. In the text, this disproportionate impact could be observed on all the characters. Shafak’s depiction of Arthur’s life is a direct critique of the patriarchal notion of progress, i.e., the Industrial Revolution. Arthur is born by the banks of the river Thames, which is an open sewer, and raised in the slums, ever mindful of the class system and oppression that followed as a consequence. His life is interconnected with both river Thames and Tigris. While the Thames existed as spatial site of oppression and escape from the rigid class structure, the river Tigris became a heterotopic space where he is free from the confines of class and is able to dedicate himself to his intellectual pursuits, regardless of class. Arthur’s life mirrors that of Thames in its transition from filth to respectability and admiration. “Sometimes he cannot tell whether he has been hypnotized into seeking the River Tigris or is simply running away from the River Thames” (Shafak, 2024, pp. 260–261). Arthur’s narrative here reflects how the rivers are not mere water bodies but ecological heterotopias that reflect the political, social, and ecological conditions of the physical space. Thames becomes a heterotopic space revealing the imperial, class-bound structure of London whose progress becomes a burden that invariably destroys the Thames and its related ecology. On the other hand, to Arthur, Tigris embodies his role as an Assyriologist and his lifelong obsession with the East. The contrast between the Thames and the Tigris demonstrates that ecological spaces do not possess fixed meanings; their significance depends upon the social and historical positions from which they are encountered.

Similarly, Zaleekhah’s narrative reveals a strong reading of the river as an ecofeminist heterotopia. She is a hydrologist estranged from her husband and moves into a houseboat on the river Thames. Yet the houseboat should not be interpreted simply as a space of liberation. Its significance lies in the alternative spatial relation it makes possible: Zaleekhah inhabits a space that is neither entirely detached from the city nor fully integrated into conventional domestic life. Her relationship with water consequently complicates fixed boundaries between public and private, scientific and personal, and ecological and social experience. Zaleekhah’s constant connection to the river, its flow, and other watery organisms reflect Neimanis’ theorization where the human body and water is constantly in a materially relational cycle and each affect the other. Her life on water and her connection to water also impact her work as a hydrologist, where she becomes aware of human-water connection and feels accountable for the state of rivers today. As a result, her work as a hydrologist is not anthropocentric, but is ecocentric and rooted in feminist politics of care. “Women are expected to be like rivers – readjusting, shapeshifting” (Shafak, 2024, p. 199). Her work on ghost streams, river pollution, and restoration while also living through isolation, loss, and depression reflect how ecological precarity has a profound impact on humans. This can also be observed in the case of Narin, a girl from the Yazidi community who was raised on the banks of Tigris in Turkey. Narin’s story traces how her and her family’s life by the river is threatened due to the building of the Ilisu Dam and the oppression they face as the travel to Iraq. The ISIS view Narin, other Yazidi women, and the river Tigris as bodies to be captured, controlled, and destroyed. Narin view Tigris as not just a river that sustains them, but a heterotopic site of memory, cultural and ancestral connection especially during the present persecution of Yazidis. The river Tigris therefore becomes an ecofeminist heterotopia where ecological injustice and gendered violence reinforce each other.

Arthur, Zaleekhah, and Narin’s narratives make it evident that rivers function ecofeminist heterotopias, or sites of gendered ecological inequality, highlighting the unequal environmental and emotional stress faced by women and other marginalized sections due to displacement, ecological violence, and the loss of cultural belonging. The novel illustrates that human lives are not disparate but deeply connected to the environment and that the harm caused to the environment is always reflected back towards people, especially women and marginalized communities. However, rivers are also juxtaposed as sites of resistance, care, and accountability towards other humans and the environment is found by realigning human-water relationship away from exploitation and towards mutual care and maintenance. When Narin’s neighbours slowly vacate their homes due to the construction of the Ilisu Dam, Narin’s grandmother delays leaving for as long as possible as an act of resistance against forced displacement and reluctance to forgo her cultural ties to the river.

By framing these riverine sites as ecofeminist heterotopias, the study seeks to understand how ecological crisis translates into continued marginality. The paper highlights the need to observe and understand ecological precarity not merely as global climate crisis but instead look at its role in social inequalities and marginalization of minorities. The study also looks at the juxtaposition of such spaces, where these sites are transformed into sites of resistance, shared memory, and cultural belonging. This highlights how ecofeminist heterotopic spaces evoke different forms of belonging and environmental awareness even during crisis.

## 6 Conclusion

This study analyses Elif Shafak’s *There Are Rivers in The Sky* (2024) through the concept of ecofeminist heterotopias to study how ecological spaces connects water, bodies, memory, displacement, and unequal relations of power. The rivers depicted in the novel do no merely function as settings, but as sites where different histories, time periods and ecological transformations coexist. Across the interconnected narratives of three characters, these riverine spaces register different experiences of industrial pollution, class inequality, gendered vulnerability, religious persecution, displacement and ecological loss.

This paper’s core contribution lies in concept of ecofeminist heterotopias. Ecofeminism draws attention to the unequal relations through which ecological harm is experienced, hydrofeminism foregrounds the material interconnectedness of bodies and water, and heterotopia explains how multiple histories and social meanings converge within particular spaces. When combined, these perspectives allow rivers to be understood as sites where ecological transformation intersects with gendered inequality, marginalization, displacement, and collective memory.

Through Arthur, Zaleekhah, and Narin, the literary text demonstrates that these experiences are differentiated and not uniform. Arthur’s relationship with the Thames foregrounds the entanglement of industrial modernity, class hierarchy and ecological transformation; Zaleekhah’s relationship with water complicates divisions between scientific and personal knowledge, domestic and public space, and ecological and social experience; and Narin’s relationship with the Tigris reveals how environmental transformation, cultural belonging and the persecution of the Yazidi community become inseparable. Hence, these waterways acquire different meanings according to the historical and social positions from which they are experienced. This difference of experiences is important as it prevents ecological vulnerability from being understood as a universal or socially neutral situation.

The study also depicts how literary representations contribute an alternate discussion on environmental inequality. In the novel, the river becomes a source of life, an archive of historical loss, a site of displacement, and a medium through which connections between bodies, places and histories persist across time. The concept of ecofeminist heterotopias thus offers a framework for examining how literary texts represent ecological spaces as locations where power and vulnerability converge, while also preserving possibilities for memory, care, cultural continuity and resistance.

Ultimately, the study argues that Shafak’s rivers challenge the separation of environmental processes from social life. Their transformation is inseparable from the transformation of the communities, histories and relationships connected to them. Reading these riverine spaces as ecofeminist heterotopias consequently reveals how ecological change is mediated by historically situated relations of gender, class, marginalization and displacement. The framework developed here may therefore be useful for further literary and interdisciplinary research examining environmental spaces not as passive backdrops to human experience, but as socially and historically constituted sites through which ecological and social relations become mutually visible.

## Data Availability

The original contributions presented in the study are included in the article/supplementary material, further inquiries can be directed to the corresponding authors.
